# Opposing Consumption Trends for Sugar-Sweetened Beverages and Plain Drinking Water: Analyses of NHANES 2011–16 Data

**DOI:** 10.3389/fnut.2020.587123

**Published:** 2020-11-16

**Authors:** Florent Vieux, Matthieu Maillot, Colin D. Rehm, Pamela Barrios, Adam Drewnowski

**Affiliations:** ^1^MS-Nutrition, 27 bld Jean Moulin, Faculté de Médecine la Timone, Laboratoire C2VN, Marseille, France; ^2^PepsiCo Inc., Purchase, NY, United States; ^3^Center for Public Health Nutrition, University of Washington, Seattle, WA, United States

**Keywords:** water tap, water bottled, sugar-sweetened beverages, NHANES 2011–2016, hydration, time trends

## Abstract

**Background:** Choosing water in place of sugar-sweetened beverages (SSB) can reduce added sugars while maintaining adequate hydration. The present goal was to examine 2011–16 time trends in SSB vs. water consumption across US population subgroups.

**Methods:** Dietary intake data for 22,716 persons aged >4 years came from two 24-h dietary recalls in successive cycles of the National Health and Examination Survey (NHANES 2011–16). Water intakes (in mL/d) from plain water (tap and bottled) and from beverages (SSB and not-SSB) were the principal outcome variables. Intakes were analyzed by age group, income to poverty ratio (IPR), and race/ethnicity. Time trends by demographics were also examined.

**Results:** SSB and water intakes followed distinct social gradients. Most SSB was consumed by Non-Hispanic Black and lower-income groups. Most tap water was consumed by Non-Hispanic White and higher-income groups. During 2011–16, water from SSB declined from 322 to 262 mL/d (*p* < 0.005), whereas plain water increased (1,011–1,144 mL/d) (*p* < 0.05). Groups aged <30 years reduced SSB consumption (*p* < 0.0001) but it was groups aged >30 years that increased drinking water (*p* < 0.001). Non-Hispanic White groups reduced SSB and increased tap water consumption. Non-Hispanic Black and lower income groups reduced SSB and increased bottled water, not tap.

**Conclusion:** The opposing time trends in SSB and water consumption were not uniform across age groups or sociodemographic strata. Only the non-Hispanic White population reduced SSB and showed a corresponding increase in tap water. Lower-income and minority groups consumed relatively little plain drinking water from the tap.

## Introduction

Choosing plain drinking water in place of sugar-sweetened beverages (SSB) is one way to maintain hydration while reducing added sugars (1, 2). Recent analyses of the three cycles of the National Health and Nutrition Examination Survey (NHANES 2011–2016) pointed to an overall decline in the consumption of sugar sweetened beverages (SSB), a finding consistent with prior reports (3, 4). This decline was offset, in part, by a corresponding increase in consumption of plain drinking water (5). Hydration was not affected, since water intakes in mL from all sources: drinking water, caloric and non-caloric beverages, and moisture from foods remained constant (5). However, given documented differences in SSB and water consumption patterns by age and demographic groups, the looked-for increases in the consumption of plain water (2) may not have occurred equally across all population strata.

First, consumption patterns for SSB and water follow very distinct socio-demographic gradients (6). SSB consumption is highest among younger adults (aged <30 years), lower income groups, and the Hispanic and non-Hispanic Black population (4, 6, 7). By contrast, plain water consumption is higher among the non-Hispanic White population and higher income groups (5–7). A social gradient also applies to tap water: its consumption was higher among groups with higher education and incomes as well as the non-Hispanic White population (5, 6).

Dietary advice to choose plain water in place of SSB may not be effective, if the beverage behaviors and consumption patterns differ across population subgroups. For example, most SSB are consumed by teenagers and young adults (5, 7) and many interventions have focused on that age group. The Healthy, Hunger-Free Kids Act of 2010 (8) required schools in the National School Lunch Program (9) to make free drinking water available during meal times (10). Schools were to allow students to have water bottles in class and to provide hydration stations (11). Based on NHANES 2011–16 data, SSB consumption has in fact declined nationally, especially among teenagers and young adults (3, 5). However, it is unclear whether this decline was accompanied by a corresponding increase in plain drinking water, tap, or bottled. Furthermore, recent data suggest that children and teenagers consume SSB and water at different times of day (12).

More SSBs are consumed by lower income and minority groups (5–7). Soda taxes were intended to reduce SSB consumption among those populations as a means to combat obesity, diabetes, and other health related problems (13). Since then, soda taxes have been credited with reducing SSB sales in selected jurisdictions, but have not been widely implemented (14). It is not clear whether the SSB were replaced with more nutrient-dense beverages or with plain drinking water. The equivalent of a 10% SSB tax led to a nonsignificant 1.9% increase in total untaxed beverage consumption (e.g., water) (14). No data on any postulated health benefits of SSB reduction on a population level are as yet available (15).

The present analyses were based on three cycles of the nationally representative NHANES 2011–2016 dietary intakes database for the US population (age ≥ 4 years) (16). The goal was to compare time trends in water and SSB consumption by age, income, and race/ethnicity. It is important to know whether the stated objectives of the US public health policies regarding replacing SSB with drinking water are being achieved across all racial/ethnic groups and socioeconomic strata.

## Methods

### NHANES 2011–16 Participant Characteristics

NHANES participants were stratified by age, race/ethnicity, and income. For primary analyses age was stratified into two categories (4–30 and ≥31 years) as SSB consumption tends to be higher among the younger age groups as compared to the older age groups. Additional analyses examined beverage consumption for more precise age groups: 4–8, 9–13, 14–18, 19–30, 31–50, 51–70 years, and >70 years. These age groups generally correspond to the age groups used by the IOM. Race/ethnicity was defined as: non-Hispanic White, non-Hispanic Black, Mexican American, other Hispanic, and other/mixed race. Family income-to-poverty ratio (IPR) is the ratio of family income to the federal poverty threshold; the cut-points for IPR were <1, 1–1.99, 2–3.49, and ≥3.5.

### NHANES 2011–16 Dietary Intakes

Consumption data for drinking water, beverages, and foods came from three cycles of the nationally representative NHANES, corresponding to years 2011–12, 2013–2014, and 2015–2016 (16). The three NHANES cycles provided a nationally representative sample of 22,716 age ≥ 4 years.

The NHANES 24-h recall uses a multi-pass method, where respondents reported the types and amounts of all food and beverages consumed in the preceding 24 h from midnight to midnight (17). The multi-pass method was conducted by a trained interviewer using a computerized interface (18). Respondents first identified a quick list of foods and beverages consumed. The time and occasion for each food item was also obtained. A more detailed cycle then recorded the amounts consumed, followed by a final probe for any often-forgotten foods (beverages, condiments). Day one interviews were conducted by trained dietary interviewers in a mobile examination center. Day two interviews were conducted by telephone some days later (19).

For children 4–5 years, dietary recall was completed entirely by a proxy respondent (i.e., parent or guardian with knowledge of the child's diet) (17). Proxy assisted interviews were conducted with children 6–11 years of age. Adolescents 12–19 years were the primary source of dietary recall data but could be assisted by an adult who had knowledge of their diet.

We used a combination of the 1-day value and the 2-day mean to make use of all available dietary data. About 90% of people had two recalls. This method included all NHANES participants, even those without a second recall. Water consumers were defined as those NHANES participants who were drinking water on day 1, 2, or both.

### Water Intakes From Water and Other Beverages

Plain drinking water included tap and bottled. Other beverages were classified as sugar sweetened beverages (SSB) and non-sugar sweetened beverages (non-SSB). Sugar sweetened beverages included regular soda, fruit drinks, sports drinks, energy drinks, presweetened ready-to-drink tea, and sweetened ready-to-drink coffee. Non-SSB included unsweetened milk and milk beverages, milk substitutes, fruit juice, diet soda, hot tea/coffee, alcoholic beverages, enhanced water, and supplemental beverages. These analyses were for water from water and SSB and non-SSB only. For example, milk consumed with cereal (i.e., not as a beverage) was not assigned to a beverage category.

The NHANES 24-h recalls for each participant provided information on the amount in grams of each food and beverage consumed (16). The present results were for mL of water derived from water and from selected beverages and not for the volume of the beverages themselves (which may not be 100% water).

### IRB and Ethical Approvals

Approvals for the conducts of the NHANES surveys had been obtained by the National Center for Health Statistics (NCHS) (20). Adult participants provided written informed consent. For children, parental/ guardian written informed consent was obtained. Children and adolescents ≥ 12 years of age provided additional written consent. All NHANES data are publicly available on the NCHS and USDA websites (16). Following University of Washington (UW) policies, analyses of public data do not involve “human subjects” and their use does not require an IRB review or an exempt determination. Such data may be used and analyzed without any involvement of the Human Subjects Division or the UW Institutional Review Board.

### Statistical Analyses

The survey-weighted mean intakes of water from SSB, other beverages (non-SSB), and drinking water in mL/day were evaluated overall and by age group, family income-to-poverty ratio, and race/ethnicity for each NHANES cycle from 2011 to 2016. First, trends in sources of hydration were compared between NHANES cycles in adults and children together and separately. For each source of hydration, a regression analysis for sample survey data was performed with water intakes from water and from beverages as dependant variable and NHANES cycles as ordinal independent one. For some analyses, water was split into tap and bottled. Tests of NHANES cycle effect over intake as well as tests for linear trend were reported. In order to assess whether previously observed trends remained in some specific strata of population, analysis was redone after stratification of the sample by detailed age classes, income to poverty ratio, and race/ethnicity. Survey-weighted means and corresponding standard errors were reported. All analyses accounted for the complex survey design of NHANES and captured nationally representative dietary behaviors of the US population between 2011 and 2016. All analyses were conducted using SAS software, version 9.4 (SAS Institute Inc., Cary NC, USA) by using SURVEYREG and SURVEYMEANS procedures, and an α level of 5% was used for all statistical tests.

## Results

### Time Trends in SSB and Water Consumption 2011–2016

Table 1 shows water intakes from water and other beverages for each NHANES cycle from 2011 to 2016. There were no significant differences in water intakes from beverages and drinking water combined between 2011 and 2016. The total amount of water was around 2,100 mL/d, evenly split between beverages and plain drinking water, tap, and bottled. No significant time trends in total water intakes were observed for the entire sample or by specific age groups.

**Table 1:** Time trends in water intakes (mL/day) from beverages including SSB and from plain drinking water, tap, and bottled (mean, standard error).

	**NHANES cycle**				
	**2011–12**	**2013–14**	**2015–16**	***p*-value**	***p*-trend**
		All >4 years	*N* = 22,716		
Beverages + water	2,108 (45)	2,077 (44)	2,114 (46)	0.8197	0.92
Beverages	1,097 (31)	1,038 (32)	970 (21)	**0.0046**	**0.0014**
SSB	322 (12)	283 (14)	262 (13)	**0.0055**	**0.0017**
Water	1,011 (33)	1,039 (30)	1,144 (38)	**0.0297**	**0.0108**
		Age 4–30 years	*N* = 10,701		
Beverages + water	1,747 (44)	1,758 (48)	1,710 (62)	0.8248	0.6303
Beverages	865 (16)	827 (32)	708 (16)	**<0.0001**	**<0.0001**
SSB	393 (13)	352 (17)	279 (16)	**<0.0001**	**<0.0001**
Water	882 (44)	930 (37)	1,001 (57)	0.2653	0.1055
		Age > 30 years	*N* = 12,015		
Beverages + water	2,336 (59)	2,275 (50)	2,364 (46)	0.4248	0.7108
Beverages	1,243 (37)	1,169 (34)	1,131 (30)	0.0753	**0.0241**
SSB	276 (19)	238 (16)	251 (14)	0.3269	0.2877
Water	1,092 (38)	1,106 (35)	1,232 (38)	**0.0195**	**0.0114**
		Females	*N* = 11,510		
Beverages + water	1,907(41)	1,917 (37)	1,937 (43)	0.8798	0.6201
Beverages	924 (24)	875 (27)	785 (23)	**0.0005**	**0.0001**
SSB	251 (13)	217 (14)	204 (17)	0.0829	**0.0397**
Water	982 (30)	1,042 (28)	1,152 (40)	**0.0059**	**0.0014**
		Males	*N* = 11,206		
Beverages + water	2,314 (67)	2,240 (52)	2,299(58)	0.6268	0.8673
Beverages	1,274 (50)	1,205 (39)	1,163 (34)	0.1962	0.0736
SSB	394 (12)	349 (17)	322 (17)	**0.0059**	**0.0023**
Water	1,040 (49)	1,035 (36)	1,136 (28)	0.1382	0.1367

For the total sample, there was a significant decline in water from beverages (−11.6%; *p* = 0.005) that was driven by a significant reduction in SSB (−18.6%; *p* = 0.0055). For the 4–30 years group, the reduction in beverages was significant (−18.2%; *p* < 0.0001) and the reductions in SSB (−29%; *p* < 0.0001) and not-SSB (*p* < 0.0001) were significant as well. The increases in plain water intakes was significant for the total sample (*p* < 0.05) and for adults >30 years (*p* < 0.05), but not for the 4–30 years age group. It appears that SSB intakes declined among people 4–30 years whereas plain water intakes increased among people >30 years.

Table 1 also shows time trends for SSB and water by sex. Reduced beverage and SSB intakes were accompanied by increased water intakes (*p* < 0.005) among females. No corresponding increase in water consumption paralleled SSB reduction among males.

### Time Trends for SSB and Water by Age Group

**Figure 1:**
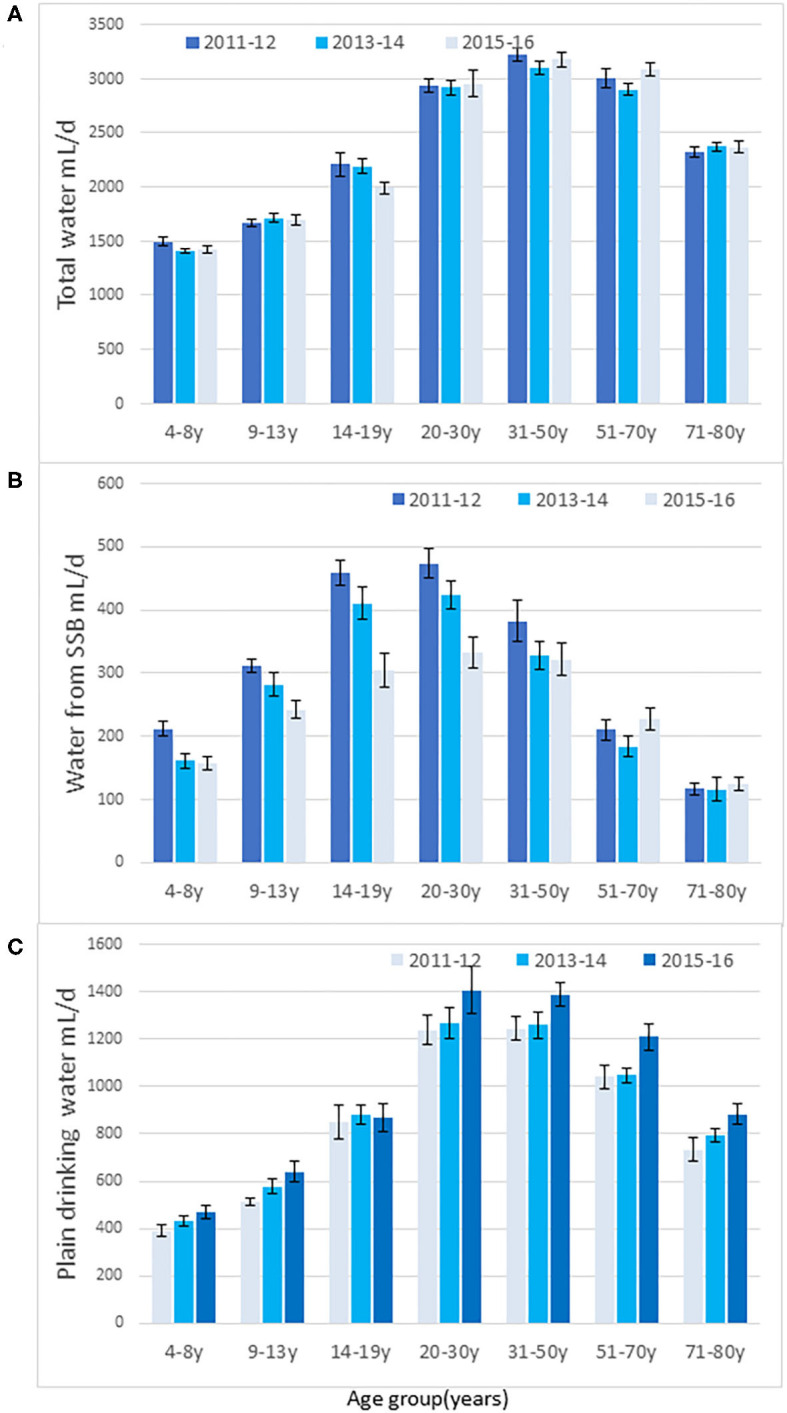
Time trends in total water intakes (mL/d) from water, beverages, and water from foods **(A)**, water from SSB **(B)**, and drinking water **(C)** by age group for each NHANES cycle. Data are means and SEM.

Figure 1 shows time trends for SSB and water by more finely differentiated age groups. First, as shown in Figure 1A total water intakes (beverages and plain drinking water) increased with age, peaked through the 31–50 years age groups, and then declined. There was no significant effect of the NHANES cycle.

Figure 1B shows that the decline in SSB was most pronounced and significant among persons aged <30 years. The decline in SSB was not significant among persons >30 years. The biggest decline (−33%) was observed among teenagers (ages 14–19 years), consistent with other reports (3, 5). However, the expected replacement of SSB with plain water in the 14–19 years age group was not observed. Rather, Figure 1C shows that increases in water consumption were more pronounced among adults over the age of 30 years (trend analyses *p* < 0.05). The increases in water intakes were significant for the 9–13 years (*p* < 0.05) and for the 51–70 years age group (*p* < 0.05).

### Time Trends for SSB and Water by IPR

**Figure 2:**
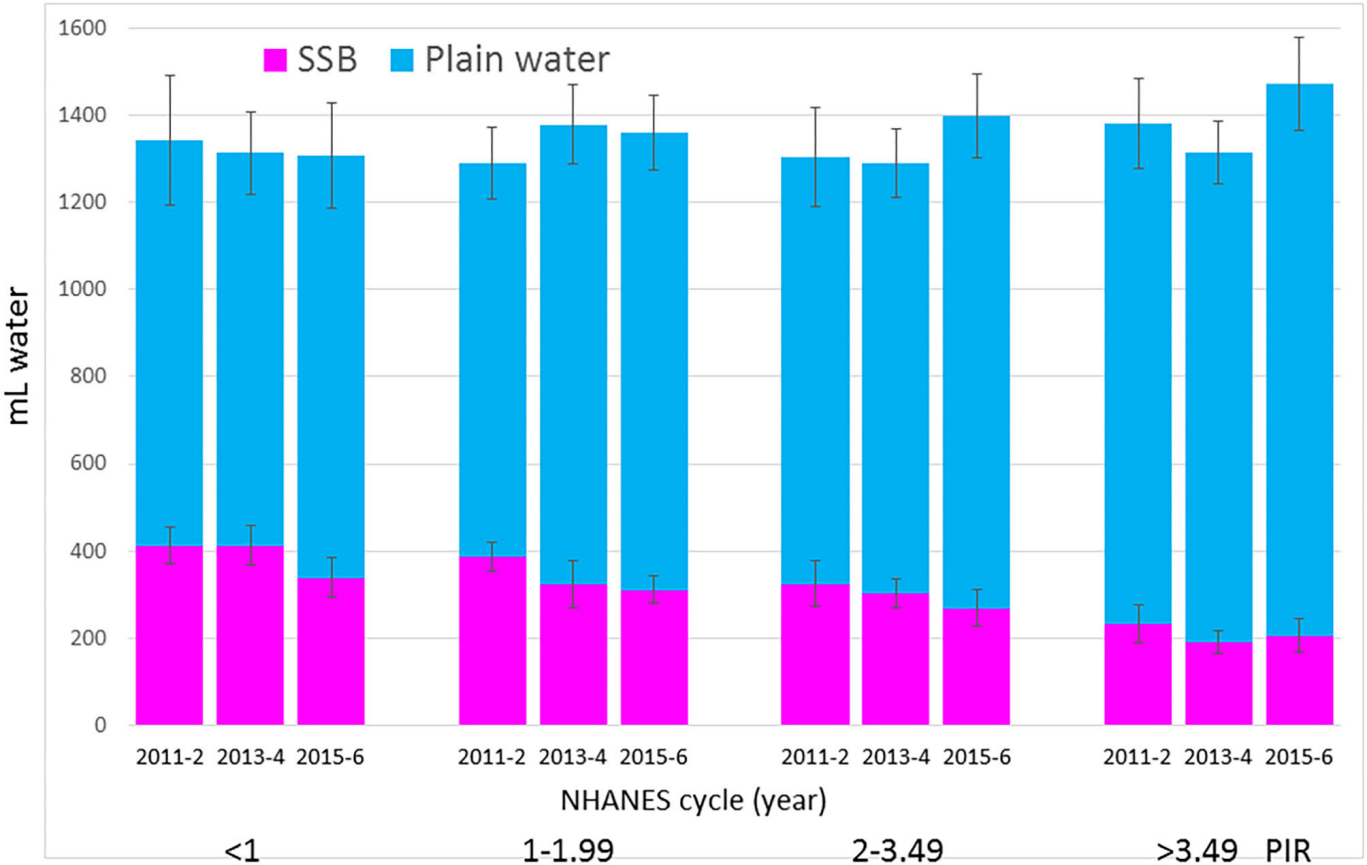
Time trends in water from SSB and drinking water (tap and bottled) by IPR and NHANES cycle. Data are means and SEM.

Figure 2 shows trends for SSB and water by family income. First, the income gradient for SSB was obtained across all NHANES cycles. Lower income groups were also the ones that reduced SSB the most (*p* < 0.03). Figure 2 also shows that the opposing income gradient for plain water also held across all NHANES cycles; higher intakes of plain drinking water were observed among the higher income groups. Those groups also showed the highest increases in water intakes.

**Figure 3:**
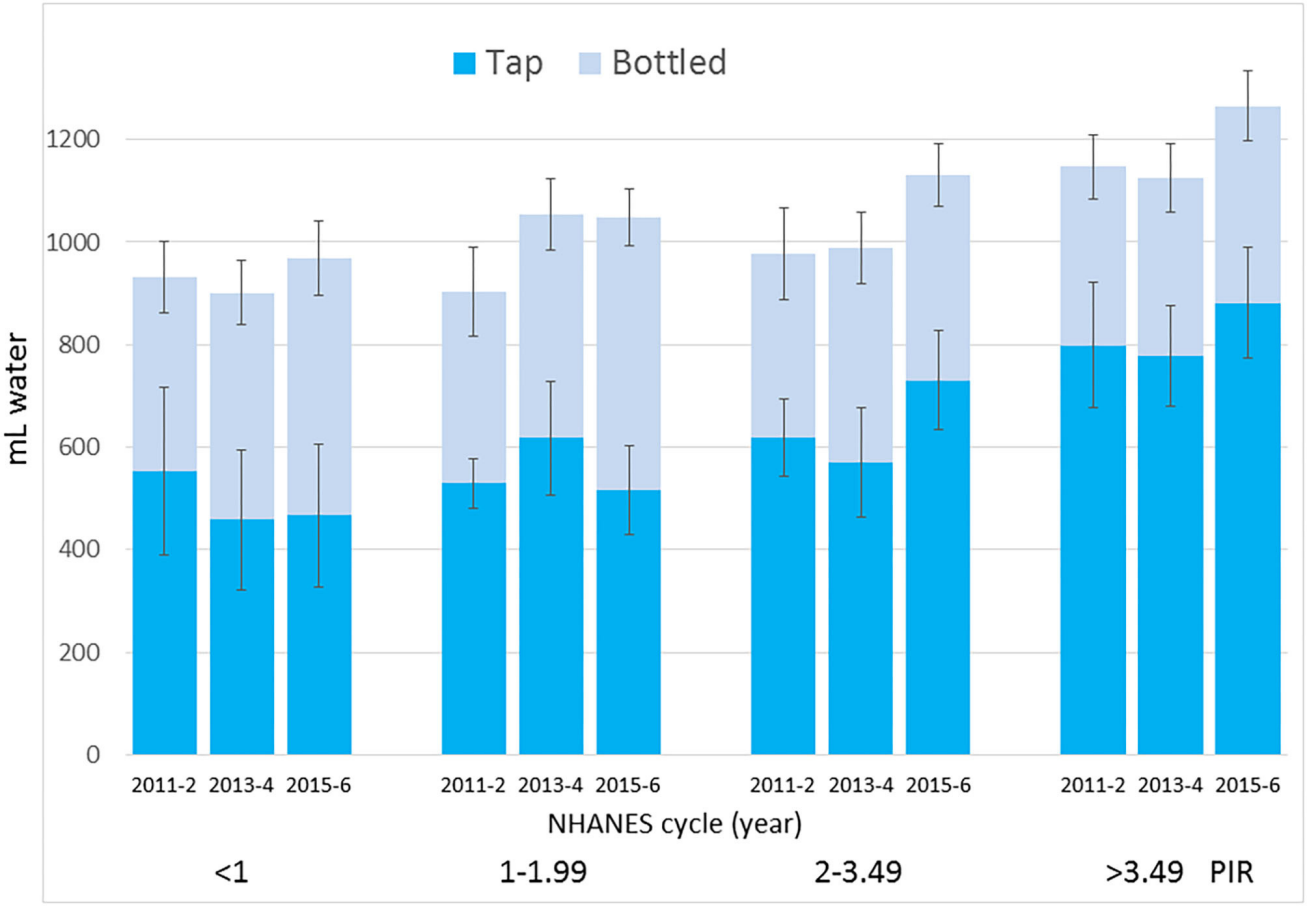
Time trends in tap water and bottled water intakes by IPR and NHANES cycle. Data are means and SEM.

Figure 3 shows the increase in tap water for the higher IPR groups. For lower IPR groups water from the tap did not increase while also showing that the lower IPR groups had a substantial and significant increase in the consumption of bottled water. It appears that the significant reduction in water from SSB among lower IPR groups was accompanied by a marked increase (144 mL/d in the IPR 1–1.99 group) in bottled water but not in tap water.

### Water From SSB and Plain Water by Race/Ethnicity

**Figure 4:**
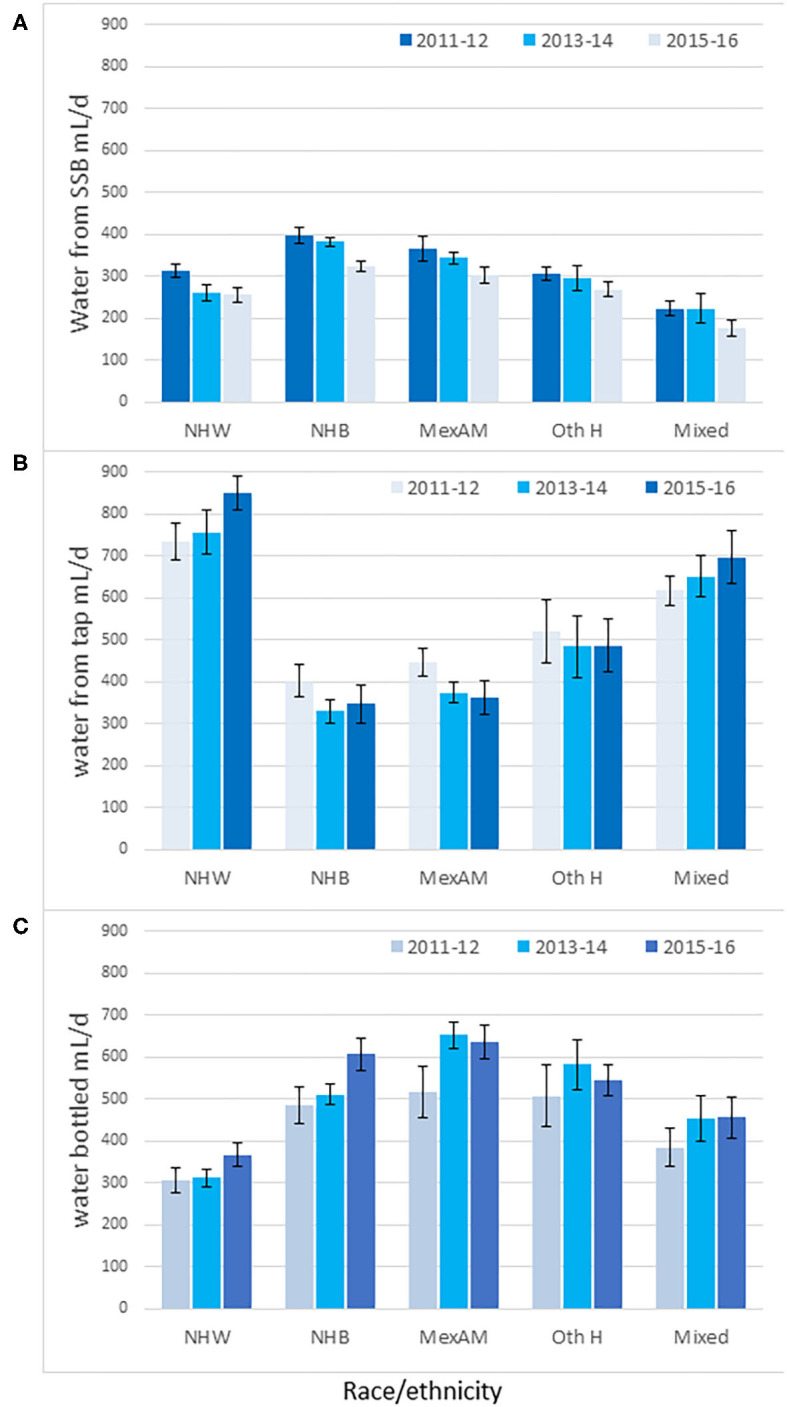
Time trends in water intakes (mL/d) from SSB **(A)**, tap **(B)** and bottled water **(C)** by race/ethnicity and NHANES cycle. Non-Hispanic White (NHW), Non-Hispanic Black (NHB), Mexican American (MexAm), other Hispanic (Oth H). Data are means and SEM.

Figure 4A shows the race/ethnicity gradient in SSB consumption. Non-Hispanic Black and Mexican American groups consumed most SSB. A significant decline in SSB was observed among non-Hispanic White (*p* < 0.05) and non-Hispanic Black groups (*p* < 0.01). No significant decline in SSB was observed for other racial/ethnic groups.

Figure 4B shows the opposing social gradient for tap water consumption. Tap water intakes were highest for non-Hispanic White and lowest for non-Hispanic Black and for Mexican American groups, whose consumption was below 400 mL/d. Analyses of whether the SSB were being replaced by plain water, tap, or bottled, pointed to some weak trends. The increase in tap water intakes was almost significant among the non-Hispanic White group (*p* for trend = 0.057) but not in any other group.

Figure 4C shows that the non-Hispanic White group consumed the least bottled water (300 mL/d). Bottled water intakes were significantly higher among the non-Hispanic Black, Mexican American, and other Hispanic groups. Bottled water intakes increased among the non-Hispanic Black population (*p* for trend <0.05) but not in any other group.

The *Y*-axes of Figure 4 are shown on the same scale to demonstrate the profound social gradients in the consumption of tap water as opposed to bottled water. Intakes of tap water among the non-Hispanic White group were higher than for the non-Hispanic Black, Mexican American, and other Hispanic groups. Conversely, intakes of bottled water among the non-Hispanic White group were lower than for the non-Hispanic Black, Mexican American, and other Hispanic groups.

## Discussion

Replacing caloric SSB with plain and non-caloric drinking water has been a priority area for public health nutrition (2). The goal of Dietary Guidelines for Americans, soda taxes, and numerous school-based initiatives is to make plain drinking water the beverage of choice (1).

The present analyses of the 2011–16 NHANES dataset confirm that the consumption of SSB in the US continues to drop (3, 5). Conversely, the consumption of plain drinking water is on the rise. However, the patterns of substitution were very different by age group, income, and race/ethnicity (21, 22).

Dietary advice to choose water *in place* of SSB may not be effective if the two beverages are normally consumed in different places, at different times of day (12) or at different eating occasions, or if habitual consumption patterns vary by age group, income, or race/ethnicity (5). SSB consumption and water intakes in the NHANES sample followed opposing income gradients. First: lower IPR groups consumed most SSB and least water; higher IPR groups consumed less SSB and more water. The non-Hispanic White population consumed least SSB, less bottled water than the other groups, and by far the most water from the tap.

The groups with the greatest reduction in SSB were not the same ones that increased the consumption of plain water. For example, the reduction in SSB was associated with younger age groups (<30 year) while the increase in plain water was associated with older age groups (>30 year). Despite federal regulations and the encouragement from schools, there was no evidence that a reduction in SSB consumption among teenagers was accompanied by a corresponding increase in plain drinking water. Clearly, the anticipated increase in plain water consumption has not been uniform across population groups.

The reduction in SSB consumption was strongest among the highest consumers, namely lower income groups. That is of interest because lower-income groups may be particularly sensitive to SSB taxes. However, those groups did not show a corresponding increase in tap water intakes. Lower IPR groups did reduce SSB and one such group (IPR 1–1.99) increased the consumption of bottled water, not tap water. Similarly, the non-Hispanic Black group, another high intake group also reduced SSB intakes and increased bottled water intakes, not tap.

There was one group that showed a decline in SSB and a corresponding increase in tap water consumption. Those were the non-Hispanic White group, who had the lowest intakes of SSB and the highest intakes of tap water. In that group, the decline in SSB was offset by an increase in tap water.

The trend away from tap water among lower income groups is troubling. It was the higher IPR groups that consumed more municipal tap water, whereas lower IPR groups consumed more bottled water. These observations are consistent with previous reports that non-Hispanic White and higher income groups consumed most tap water (6); Mexican Americans drank the most bottled water and the least tap water (5, 6).

This could be due to the “Flint effect,” that is the perception that tap water is safe to drink only in affluent neighborhoods (22, 23). One paper (23) notes that the mistrust of tap water was one reason for SSB consumption. The odds of consuming ≥1 SSB/d among Hispanic respondents who mistrusted their local tap water was twice that of those who did not (23). As the quality of tap water in lower income areas becomes problematic (22, 24), the consumption of bottled water is on the rise among lower income groups and the non-Hispanic Black group.

Many initiatives have focused on tap water describing it as “the perfect, no-cost, no-calorie beverage, and it comes right out of the kitchen tap” (25). Providing tap water to children is another initiative (26, 27).

Making water the national beverage of choice (DGAs) is a strategy that needs to be more sensitive to the quality of the local water supply and to community resources, wants, and needs.

The present analyses had limitations. First, the NHANES data are based on self-report and are subject to random and systematic reporting errors. A 24-h recall may systematically underestimate water and other beverage intake, especially outside of meals since it is very difficult for individuals to remember exactly how much tap water they had outside of meals. The present estimates, based on a combination of day 1 and 2 dietary recalls may have been affected by differences in data collection procedures across the 2 days. Fluid-specific records, used in smaller scale studies, may provide higher quality data. The use of proxy respondents for children ages 4–5 years and proxy assisted interviews for children 6–11 make the collection of accurate data especially challenging. The two days of dietary recalls used different methods to collect the data, which may affect the estimates of water consumption. However, the NHANES has the advantage of being based on a large, nationally representative population sample. The NHANES dataset forms the basis for dietary surveillance in the US.

## Conclusion

Reduced intakes of SSB among non-Hispanic White groups and among females were accompanied by a parallel increase in plain water intakes. Less consistent trends were observed among other population subgroups. Non-Hispanic Black and lower income groups consumed more bottled water. Non-Hispanic While and higher-income groups consumed more plain water from the tap. Successful implementation of Dietary Guidelines to choose water over SSB may depend on population beverage habits. Further research is needed to understand how these changes are being made and whether further interventions may be necessary.

## Data Availability Statement

Data used in the study is publicly available through the NHANES database (available at https://wwwn.cdc.gov/nchs/nhanes/continuousnhanes/default.aspx).

## Ethics Statement

The studies involving human participants were reviewed and approved by https://www.cdc.gov/nchs/nhanes/about_nhanes.htm (accessed October 9, 2019). The patients/participants provided their written informed consent to participate in this study.

## Author Contributions

All authors (FV, MM, CR, PB, and AD) conceptualized study design, formulated analytical questions, and contributed to the manuscript preparation. CR created the dataset, while FV and MM performed the principal analyses. AD acted as lead writer of the paper. All authors (FV, MM, CR, PB, and AD) reviewed and approved the final manuscript.

## Conflict of Interest

FV and MM are employees of MS-Nutrition, a start-up. PB and CR are employed by PepsiCo Inc. AD has received contracts, consulting fees and honoraria from entities both public and private with an interest in nutrient profiling of beverages and in beverage consumption, including manufacturers and distributors of both SSB and bottled water such as PepsiCo, Nestlé, and Danone. The views expressed in this work are those of the authors and do not necessarily reflect the position or policy of PepsiCo Inc.
