# Supplementary material for: Opposing Consumption Trends for Sugar-Sweetened Beverages and Plain Drinking Water: Analyses of NHANES 2011–16 Data
Source: Front Nutr. 2020 Nov 16;7:587123. doi: 10.3389/fnut.2020.587123 (PMC7701252; doi:10.3389/fnut.2020.587123)
Supplement: Supplementary file 1 [file Table_1.DOCX]

**Florent Vieux, Matthieu Maillot, Colin D. Rehm, Pamela Barrios, Adam Drewnowski. Opposing consumption trends for sugar-sweetened beverages and plain drinking water : Analyses of NHANES 2011-16 data**

**Supplemental Table S1. Time trends in water intakes across NHANES cycle for beverages by category. Dependent variable is water intake (mL) from beverages.**

|  | NHANES cycle (year) | | | |  |
| --- | --- | --- | --- | --- | --- |
| **Whole population** | 2011-12 | 2013-14 | 2015-16 | Test | Test trend |
| **Water (mL)** | | | | | |
| Milk | 87.75(6.2) | 78.08(3.51) | 71.91(5.56) | 0,1729 | 0.0633 |
| Milk substitute | 2.91(0.35) | 2.85(0.46) | 2.81(0.48) | 0,9864 | 0.8724 |
| Citrus juices | 34.18(3.61) | 27.9(0.99) | 26.13(1.66) | 0,1379 | 0.0487 |
| Non citrus juices | 28.3(1.83) | 25.35(1.67) | 22.79(1.54) | 0,0801 | 0.0257 |
| Soda regular | 166.9(9.09) | 151.52(9.21) | 129.45(6.05) | 0,0035 | 0.0013 |
| Soda. diet | 88.59(7.21) | 81.36(5.81) | 61.8(6.81) | 0,0237 | 0.0096 |
| Ready to drink tea | 104.8(10.31) | 107.04(7.32) | 112.6(9.43) | 0,8394 | 0.5793 |
| Ready to drink coffee | 5.96(0.93) | 14.82(1.94) | 14.01(2.35) | <.0001 | 0.0026 |
| Fruit drinks | 68.52(3.91) | 50.07(2.51) | 43.25(2.05) | <.0001 | <0.0001 |
| Sports drinks | 29.54(4.12) | 33.76(3.55) | 26.65(3.8) | 0,3943 | 0.6088 |
| Energy drinks | 6.62(0.88) | 8.77(1.32) | 8.64(1.55) | 0,3019 | 0.2646 |
| Hot Coffee/tea | 301.82(16.03) | 268.65(16.73) | 276.99(14.28) | 0,3274 | 0.2532 |
| Alcohol | 133.2(11.45) | 130.23(12.92) | 123.88(8.54) | 0,7926 | 0.5178 |
| Enhanced water | 19.26(3.02) | 24.34(2.78) | 17.41(2.23) | 0,158 | 0.6249 |
| Other beverages | 9.76(2.31) | 19.5(1.56) | 18.04(1.58) | 0,0035 | 0.0049 |
| Supp | 8.72(1.09) | 13.57(2.02) | 13.48(1.9) | 0,0316 | 0.0348 |
